# Identical tau filaments in subacute sclerosing panencephalitis and chronic traumatic encephalopathy

**DOI:** 10.1186/s40478-023-01565-2

**Published:** 2023-05-05

**Authors:** Chao Qi, Masato Hasegawa, Masaki Takao, Motoko Sakai, Mayasuki Sasaki, Masashi Mizutani, Akio Akagi, Yasushi Iwasaki, Hiroaki Miyahara, Mari Yoshida, Sjors H. W. Scheres, Michel Goedert

**Affiliations:** 1 Medical Research Council Laboratory of Molecular Biology, Cambridge, UK; 2 Department of Brain and Neuroscience, Tokyo Metropolitan Institute of Medical Science, Tokyo, Japan; 3 Department of Clinical Laboratory and Internal Medicine, National Center of Neurology and Psychiatry, Tokyo, Japan; 4 Department of Neurology and Brain Bank, Mihara Memorial Hospital, Isesaki, Japan; 5 Department of Neurology, National Hospital Organization Suzuka National Hospital, Suzuka, Mie Japan; 6 Department of Child Neurology, Center of Neurology and Psychiatry, Tokyo, Japan; 7 Department of Neuropathology, Institute for Medical Science of Aging, Aichi Medical University, Nagakute, Aichi Japan

**Keywords:** Tau, Subacute sclerosing panencephalitis, Chronic traumatic encephalopathy, Inflammation, Electron cryo-microscopy

## Abstract

Subacute sclerosing panencephalitis (SSPE) occurs in some individuals after measles infection, following a symptom-free period of several years. It resembles chronic traumatic encephalopathy (CTE), which happens after repetitive head impacts or exposure to blast waves, following a symptom-free period. As in CTE, the neurofibrillary changes of SSPE are concentrated in superficial cortical layers. Here we used electron cryo-microscopy (cryo-EM) of tau filaments from two cases of SSPE to show that the tau folds of SSPE and CTE are identical. Two types of filaments were each made of two identical protofilaments with an extra density in the β-helix region. Like in CTE, the vast majority of tau filaments were Type I, with a minority of Type II filaments. These findings suggest that the CTE tau fold can be caused by different environmental insults, which may be linked by inflammatory changes.

**Supplementary Information:**

The online version contains supplementary material available at 10.1186/s40478-023-01565-2.

## Introduction

Subacute sclerosing panencephalitis (SSPE) is a fatal disorder of the central nervous system that occurs following infection with measles virus and manifests itself after a symptom-free period of several years [1]. It occurs in approximately 1 in 75,000 cases of measles [2]. The neuropathology of SSPE is characterized by severe nerve cell loss, demyelination, perivascular lymphocytic infiltrations and viral intranuclear inclusion bodies. By silver staining, abundant neurofibrillary tangles are present in cerebral cortex and other brain regions in a proportion of cases [3, 4]. They have been reported to be made of paired helical filaments like those from Alzheimer’s disease brains [5, 6] and stain for abnormally phosphorylated tau and ubiquitin [7]. Tangle-bearing cases of SSPE have mostly a long disease duration [8] and in a recent immunohistochemical study, tau pathology was found in all cases of SSPE [9].

Tauopathy has been inferred to result from diffuse brain inflammation triggered by infection with measles virus and not from a direct effect of the virus [9]. Neurofibrillary tangles of SSPE stain with antibodies specific for 3R and 4R tau, like the tau inclusions of primary age-related tauopathy (PART), Alzheimer’s disease (AD) and chronic traumatic encephalopathy (CTE) [10]. Unlike PART and AD, but like CTE, the neurofibrillary tangles of SSPE are present in superficial cortical layers [9, 11].

We previously used electron cryo-microscopy (cryo-EM) to determine the atomic structures of tau filaments from a number of neurodegenerative conditions, which has resulted in a structure-based classification of tauopathies [12]. We showed that the tau filament folds of 3R + 4R tauopathies separate into two groups, the first of which is formed by PART, AD, and familial British and Danish dementias (FBD and FDD), and the second by CTE. The neurofibrillary pathology associated with some cases of Gerstmann-Sträussler-Scheinker disease (GSS) also belongs to the first group [13]. We now report that the structures of tau filaments from two cases of SSPE are identical to those of CTE. This suggests that the CTE tau fold can form in response to different environmental insults, which may be linked by inflammatory changes.

## Materials and methods

### Clinical history and neuropathology

We determined the cryo-EM structures of tau filaments from the frontal cortex of two individuals with SSPE. There was no history of head injury. Case 1 was a male who developed measles when 1.5 years old; at age 8, he developed a speech disturbance, as well as eating and walking difficulties. He was diagnosed with SSPE based on clinical presentation, a history of measles infection and characteristic electro-encephalogram and cerebrospinal fluid abnormalities. Despite intensive antiviral treatment, his condition worsened progressively and he died aged 42, after having been on mechanical ventilation for 11 years. The brain was severely atrophic with a weight of 439 g. Neuronal rarefaction was extensive in cerebrum, brainstem and cerebellum and there was a severe loss of myelinated nerve fibres. Tau-immunoreactive NFTs showed a wide distribution and were present in layers II and III of the cerebral cortex. The clinicopathological characteristics of SSPE case 2 have been described [case 4 in [9]]. Briefly, this was a male who developed measles at the age of 0.8 year; at age 22, he developed convulsions, myoclonus and parkinsonism. He was diagnosed with SSPE based on the neurological findings, a history of measles infection, periodic synchronous discharge on electro-encephalogram and cerebrospinal fluid abnormalities. Intensive antiviral treatment failed to improve his symptoms, he became bedridden and died aged 41. The brain was severly atrophic with a weight of 735 g. Pathological examination revealed the presence of marked brain atrophy with nerve cell loss and gliosis in cerebral cortex, basal ganglia, thalamus and hippocampus, which were associated with severe white matter atrophy [9]. Tau immunoreactive NFTs showed a broad distribution, in particular in superficial layers II and III of the cerebral cortex, in oculomotor nuclei and in the locus coeruleus.

### Extraction of tau filaments

Sarkosyl-insoluble material was extracted from the frontal cortex of cases 1 and 2 of SSPE, as described [14], with minor modifications. Briefly, tissues were homogenized with a Polytron in 40 vol (w/v) extraction buffer consisting of 10 mM Tris–HCl, pH 7.4, 0.8 M NaCl, 10% sucrose and 1 mM EGTA. Homogenates were brought to 2% sarkosyl and incubated for 30 min at 37 °C. Following a 10 min centrifugation at 27,000 *g*, the supernatants were spun at 257,000 *g* for 30 min. Pellets were resuspended in 2 ml extraction buffer containing 1% sarkosyl and centrifuged at 166,000 *g* for 20 min. The resulting pellets were resuspended in 100 µl phosphate-buffered saline (PBS) and used for subsequent analyses.

### Immunolabelling and histology

Immunogold negative-stain electron microscopy and immunoblotting were carried out as described [15]. For immunoelectron microscopy, the samples were applied onto collodion membrane-applied mesh, blocked with 0.3% gelatin, incubated with AT8 (1:100, Innogenetics 90,206) for 1 h at 37 °C, followed by a 1 h incubation at 37 °C with 10 nm gold-labelled secondary antibody (1:50) and staining with 2% phosphotungstate. For immunoblotting, samples were run on 5–20% gradient gels (Fuji Film). Proteins were then transferred to a polyvinylidene difluoride membrane and incubated with the following primary antibodies overnight at room temperature: Tau N (1:1000, Cosmobio TIP-TAU-P-03), AT8 (1:500), RD3 (1:500, Millipore 05-803), RD4 (1:500, Millipore 05-804), anti-4R (1:1000, BioLegend MMS-5020), Tau354-369 (1:1000, Millipore ABN2178-100UL) and T46 (1:1000, Thermo Fisher Scientific 13-6400). Following washing in PBS, the membranes were incubated with biotinylated anti-mouse or anti-rabbit secondary antibody (Vector, 1:500) for 1 h at room temperature, followed by a 30 min incubation with avidin–biotin complex and colour development using NiCl-enhanced diaminobenzidine as substrate. Histology and immunohistochemistry were carried out as described [16]. Brain sections were 8 µm thick and were counterstained with haematoxylin. Primary antibodies were: RD3 (1:1000); anti-4R (1:1000); AT8 (1:300); anti-measles virus fusion protein antibody (1:1000, Funakoshi bs-0886R); Iba-1 (1:2000, 019-19741); CD3 (1:1000, Novocastra NCL-1-CD3-565).

### Electron cryo-microscopy: sample preparation and data collection

Extracted tau filaments were centrifuged at 3000 *g* for 1 min and applied to UltrAuFoil cryo-EM grids [17], which were glow-discharged with an Edwards (S150B) sputter coater at 30 mA for 1 min. Aliquots of 3 µl were applied to the glow-discharged grids, blotted with filter paper and plunge-frozen into liquid ethane using a Vitrobot Mark IV (Thermo Fisher Scientific) at 100% humidity and 4 °C. Cryo-EM images were collected on a Titan Krios electron microscope (Thermo Fisher Scientific) operated at 300 kV and equipped with a Falcon-4 direct electron detector. Images were recorded during 6 s exposures in EER (electron event representation) format [18] with a total dose of 40 electrons per A^2^ and a pixel size of 0.824 Å.

### Electron cryo-microscopy: image processing

Image processing was performed using RELION-4.0 [19, 20], unless indicated otherwise. Raw movie frames were gain-corrected, aligned, dose-weighted and summed into a single micrograph. Contrast transfer function (CTF) parameters were estimated using CTFFIND-4.1 [21]. Filaments were picked manually and segments extracted initially with a box size of 1024 pixels. 2D classification was used to remove suboptimal segments and separate Type I from Type II filaments. Selected class averages for Type I and Type II filaments were then re-extracted with a box size of 400 (for SSPE case 1) or 300 (for SSPE case 2) pixels. Initial models were generated de novo from 2D class averages using *relion_helix_inimodel2d* [22]. Helical twist and rise were optimised during 3D auto-refinement. Bayesian polishing and CTF refinement [23] were used to improve the resolution of reconstructions of Type I filaments. Final maps were sharpened using standard post-processing procedures in RELION and their resolutions were calculated based on the Fourier shell correlation (FSC) between two independently refined half-maps at 0.143 [24]. Helical symmetry was imposed on the post-processed maps using the *relion_helix_toolbox* program [25].

### Model building and refinement

Atomic models of published CTE filament structures [26] (PDB:6NWP; PDB:6NWQ) were docked manually in the density using Coot [27]. Model refinements were performed using *Servalcat* [28] and Refmac5 [29, 30]. Models were validated with MolProbity [31]. Figures were prepared with ChimeraX [32] and Pymol [33]. Further details of data acquisition and image processing are given in Additional file 1: Table S1 and Figure S1.

## Results

For cryo-EM, we extracted tau filaments from the frontal cortex of two cases of SSPE. Both individuals had measles as children, with the clinical picture of SSPE manifesting itself following a number of symptom-free years.

**Fig. 1:**
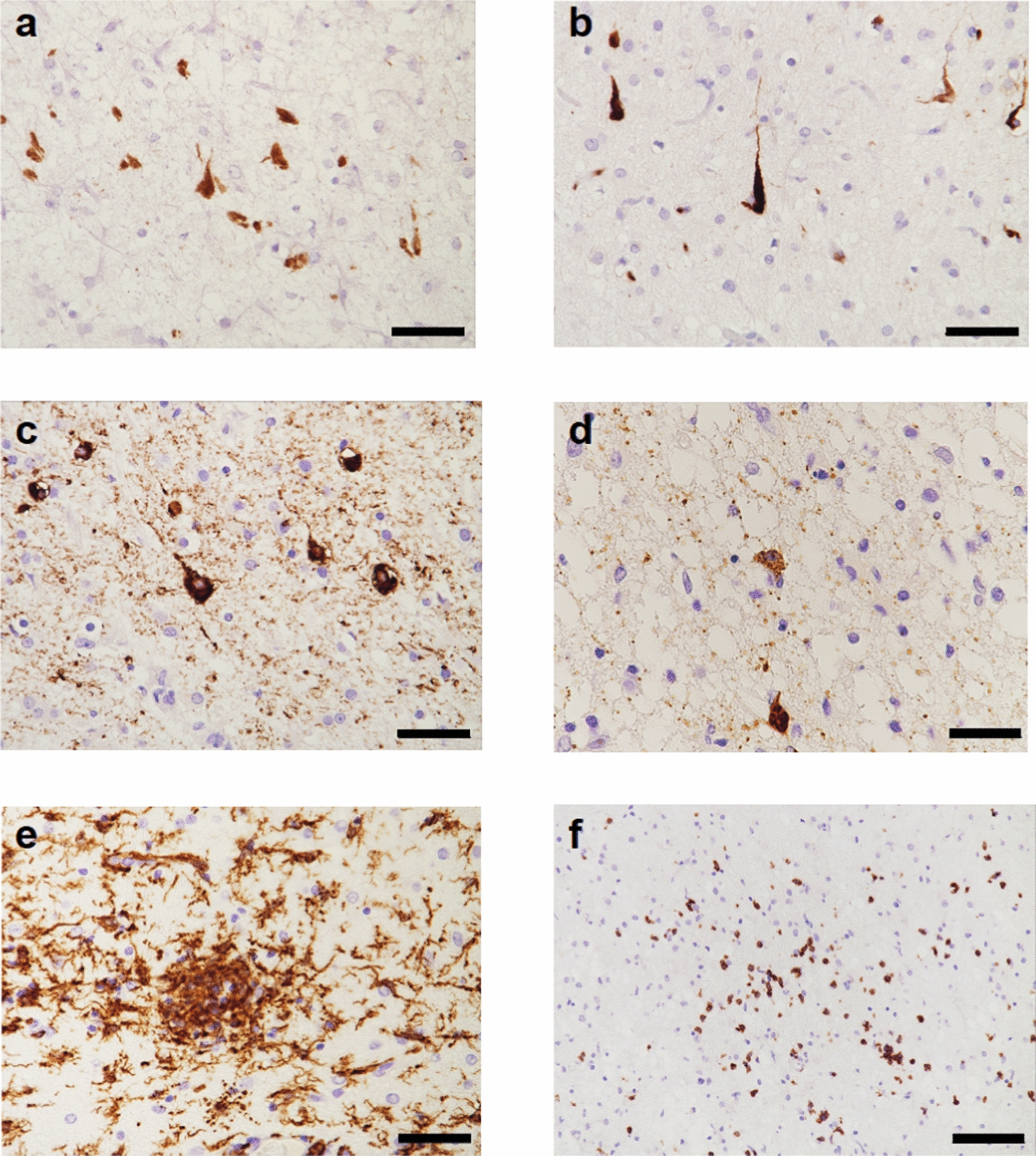
Frontal cortex from SSPE case 1: Immunohistochemical characterisation of tau inclusions and inflammatory changes. **a** RD3 (specific for 3R tau)-immunoreactive nerve cells and neuropil threads. **b** RD4 (specific for 4R tau)-immunoreactive nerve cells and neuropil threads. **c** AT8 (specific for pS202 and T205 tau)-immunoreactive nerve cells and neuropil threads. **d** Antibody against measles virus shows neuronal staining. **e**, Iba-1-immunoreactive microglial cells. **f** CD3-immunoreactive lymphocytes. Many tau inclusions were found in layers II and III. Scale bars: **a**–**e**, 50 µm; f, 100 µm.

In SSPE case 1, immunohistochemistry with anti-tau antibodies showed abundant neurofibrillary tangles that were stained by anti-tau antibodies specific for 3R tau, 4R tau and phospho-tau (AT8) (Fig. 1). Neurons and glial cells with intranuclear inclusion bodies were observed using an antibody against measles virus. Microglial cells were activated, as shown by Iba-1 staining; the same was true of cytotoxic and T helper lymphocytes, as evidenced by CD3 staining (Fig. 1). Similar abnormalities have been described in SSPE case 2 [9].

**Fig. 2:**
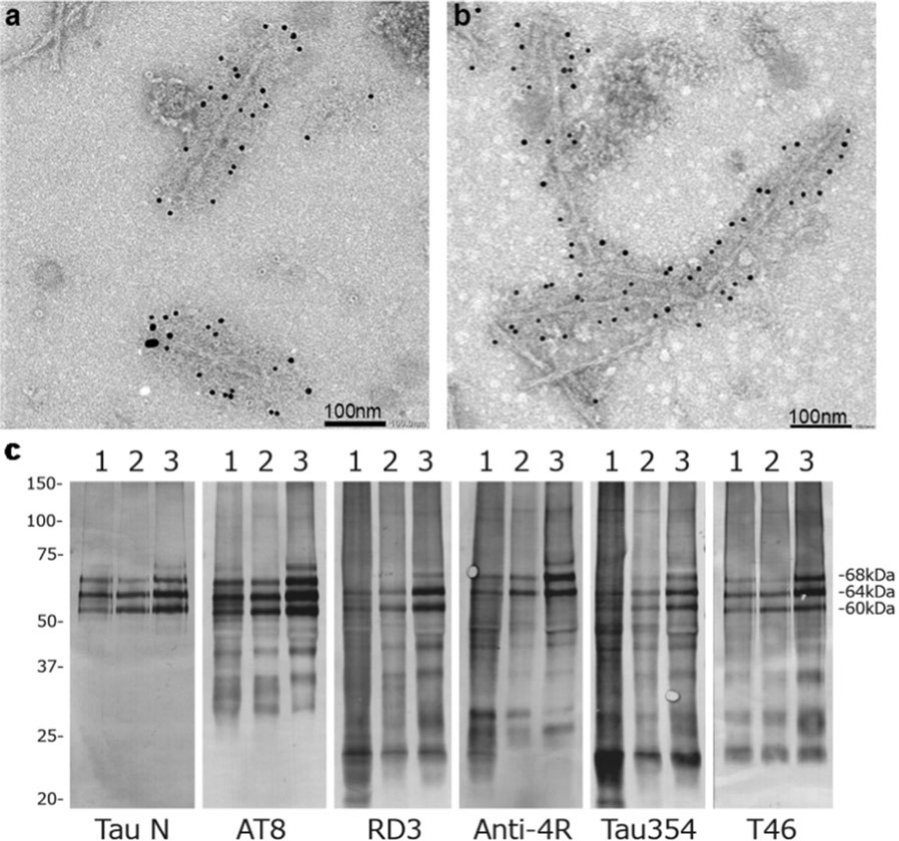
Immunolabelling and immunoblotting of tau filaments from SSPE. **a**, **b** Immunoelectron microscopy of filaments from SSPE cases 1 (**a**) and 2 (**b**) using anti-tau antibody AT8. **c** Immunoblotting of sarkosyl-insoluble fractions using anti-tau antibodies: Tau N; AT8; RD3; Anti-4R; Tau354; T46. Lanes: 1, SSPE case 1; 2, SSPE case 2; 3, AD

Filaments from the sarkosyl-insoluble fractions were decorated by anti-tau antibodies and gave the same bands on Western blots as those from AD brains (Fig. 2). It has previously been shown that the bands of sarkosyl-insoluble tau from AD are identical to those from CTE [34]. The observed bands indicate that the filaments of SSPE cases 1 and 2 are made of all six tau isoforms in a hyperphosphorylated state, consistent with previous results [9].

**Fig. 3:**
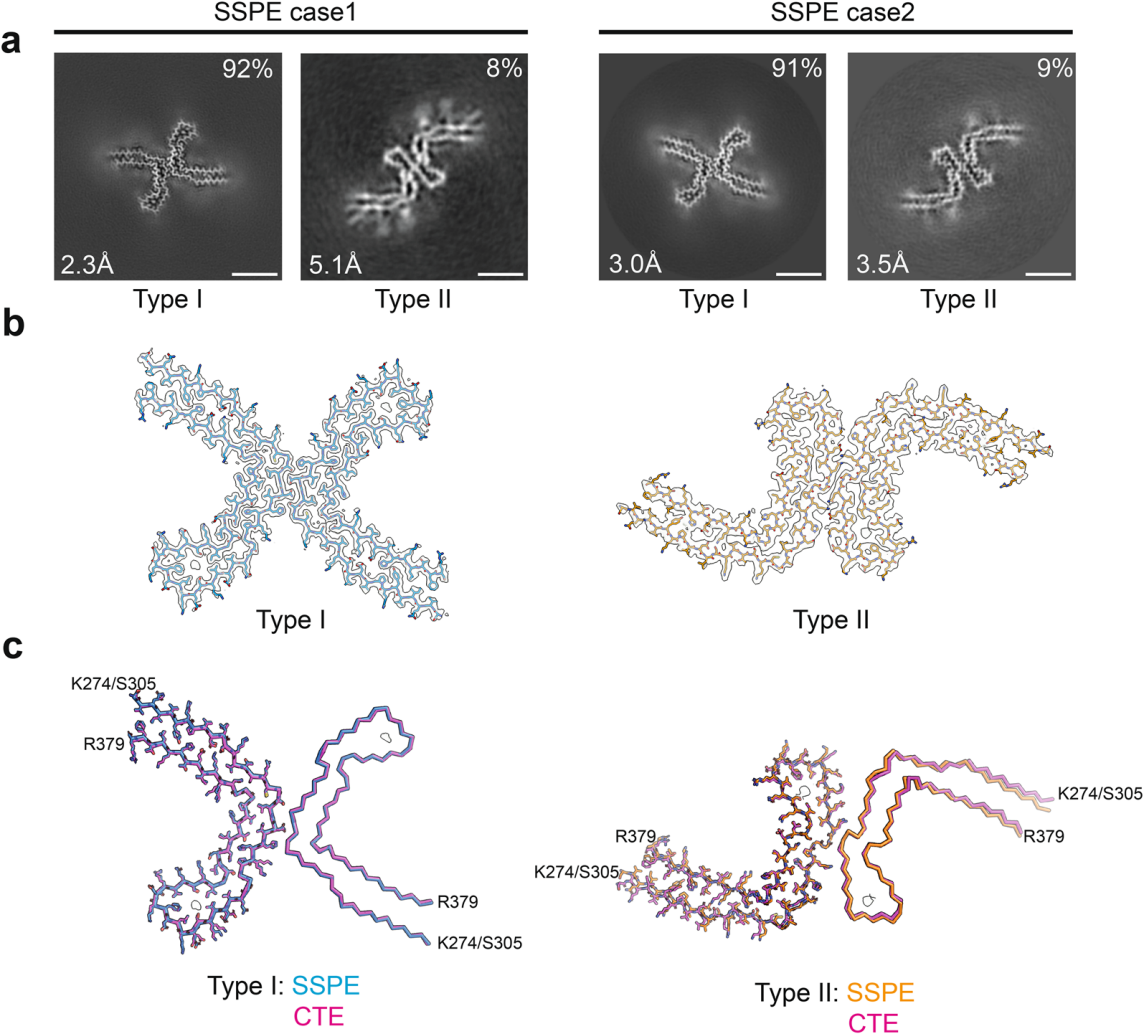
Cryo-EM cross-sections and structures of tau filaments from SSPE. **a** Cross-sections through the cryo-EM reconstructions, perpendicular to the helical thickness and with a projected thickness of approximately one rung, are shown for SSPE cases 1 and 2. A majority of Type I and a minority of Type II tau filaments are each made of two copies of a single protofilament arranged in different ways (ultrastructural polymorphs). Resolutions and filament percentages: SSPE case 1: Type I CTE filament 2.3 Å, 92%; Type II CTE filament 5.1 Å, 8%. SSPE case 2: Type I CTE filament 3.0 Å, 91%; Type II CTE filament 3.5 Å, 9%. Scale bar, 5 nm. **b** Cryo-EM density maps (grey transparent) of SSPE Type I and Type II tau filaments and the atomic models coloured blue (Type I) and orange (Type II). **c** SSPE Type I (blue) and Type II (orange) filaments overlaid with CTE Type I (magenta) and CTE Type II (magenta) filaments. The filament core extends from tau residues K274/S305-R379

By cryo-EM, we show that the CTE fold of assembled tau is characteristic of SSPE cases 1 and 2 (Fig. 3; Additional file 1: Figure S1). As in the CTE fold, two types of tau filaments were present, each with an unknown, internal density in the β-helix of the structured core. Type I and Type II filaments are both made of two identical protofilaments, with different interprotofilament packing, i.e. type I and type II CTE filaments are ultrastructural polymorphs. Type I comprised more than 90% of the observed filaments and Type II less than 10%, similar to what has been observed in CTE [26].

## Discussion

SSPE and CTE share a long interval between the primary insult (measles virus infection or repetitive head impacts and blast waves) and the appearance of clinical symptoms. Like AD and CTE, tangle-bearing cases of SSPE are characterised by the presence of abundant filamentous inclusions made of all six brain tau isoforms. Unlike AD, CTE and tangle-bearing cases of SSPE share the formation of abundant tau inclusions in cortical layers II and III [9, 11]. We previously showed that the CTE fold differs from the Alzheimer tau fold by adopting a more open conformation of the β-helix region, which contains an internal density of unknown identity [26]. In the presence of NaCl, recombinant tau comprising amino acids 297–391 assembles into filaments with the CTE fold, but in its absence, the Alzheimer tau fold forms [35].

We now find that the tau fold of SSPE is identical to that of CTE. As in CTE, two types of filaments, each made of two identical protofilaments, were present in SSPE cases 1 and 2. Western blots of sarkosyl-insoluble tau fractions indicate that these filaments are made of all six (3R + 4R) brain tau isoforms [12, 13, 26, 36]. For 3R + 4R tauopathies, one protofilament fold (the Alzheimer fold) is found in PART, AD, GSS, FBD and FDD, and the second protofilament fold (the CTE fold) is found in CTE. The present findings show that SSPE is a second example of a 3R + 4R tauopathy with the CTE fold. It remains to be seen if other conditions with tau inclusions in cortical layers II and III also share the CTE fold.

Inflammation may be what CTE and SSPE have in common. In SSPE case 2, extensive inflammatory changes have been described, with perivascular lymphocyte infiltration, aggregates of hypertrophic astrocytes and activated microglia [9]. Here we show that the frontal cortex from SSPE case 1 also exhibited microglial activation and lymphocyte infiltration. Immunoreactivity for measles virus was present in both cases of SSPE, even though they had undergone antiviral treatment. In untreated cases of long disease duration, measles virus was detected in cases with neurofibrillary lesions [37]. In treated cases, the detection frequency of measles virus was decreased, even though neurofibrillary pathology was unaffected, suggesting that antiviral therapies may not be able to suppress the progression of tauopathy following SSPE [9]. It remains to be seen if tau inclusions influence the clinical picture of SSPE. Inflammatory changes also occur in CTE, where microglial cell activation is believed to increase tau pathology and the presence of abundant CD68-positive microglial cells has been demonstrated [38]. Moreover, translocator protein (TSPO) positron emission tomography ligands for activated microglia have shown increased signal in retired American football players who are at risk for CTE [39].

It is unclear how inflammation and microglial cell activation affect tau assembly. Microglial cell activation has been reported to promote tau assembly in mice [40–42] and it also characterizes neurodegenerative diseases with filamentous tau pathology other than SSPE and CTE, the most studied of which is AD [43, 44]. More work is required to identify the links between neuroinflammation and tau assembly.

## Supplementary Information

**Additional file 1.** Supplementary information: Table S1 and Figure S1.

## Data Availability

Cryo-EM maps have been deposited in the Electron Microscopy Data Bank (EMDB) with the accession numbers EMD-16532, EMD-16535. Corresponding refined atomic models have been deposited in the Protein Data Bank (PDB) under accession numbers 8CAQ, 8CAX. Please address requests for materials to the corresponding authors.
