# Supplementary material for: Identical tau filaments in subacute sclerosing panencephalitis and chronic traumatic encephalopathy
Source: Acta Neuropathol Commun. 2023 May 5;11:74. doi: 10.1186/s40478-023-01565-2 (PMC10161654; doi:10.1186/s40478-023-01565-2)
Supplement: Supplementary file 1 — Additional file 1. Supplementary information: Table S1 and Figure S1. [file 40478_2023_1565_MOESM1_ESM.docx]

**SUPPLEMENTARY INFORMATION**

**IDENTICAL TAU FILAMENTS IN SUBACUTE SCLEROSING PANENCEPHALITIS AND CHRONIC TRAUMATIC ENCEPHALOPATHY**

Chao Qi^1+,^ Masato Hasegawa^2+^, Masaki Takao^3,4^, Motoko Sakai^5^, Mayasuki Sasaki^6^, Masashi Mizutani^3^, Akio Akagi^7^, Yasushi Iwasaki^7^, Hiroaki Miyahara^7^, Mari Yoshida^7^, Sjors H.W. Scheres^1*^, Michel Goedert^1*^

1. Medical Research Council Laboratory of Molecular Biology, Cambridge, UK
2. Department of Brain and Neuroscience, Tokyo Metropolitan Institute of Medical Science, Tokyo, Japan
3. Department of Clinical Laboratory and Internal Medicine, National Center of Neurology and Psychiatry, Tokyo, Japan
4. Department of Neurology and Brain Bank, Mihara Memorial Hospital, Isesaki, Japan
5. Department of Neurology, National Hospital Organization Suzuka National Hospital, Mie, Japan
6. Department of Child Neurology, Center of Neurology and Psychiatry, Tokyo, Japan
7. Department of Neuropathology, Institute for Medical Science of Aging, Aichi Medical University, Aichi, Japan

^+^Equal contributions

^*^Corresponding authors. scheres@mrc-lmb.cam.uk; [mg@mrc-lmb.cam.ac.uk](mailto:mg@mrc-lmb.cam.ac.uk)

**Table S1.**

**Cryo-EM data collection, refinement and validation statistics.**

|  | SSPE_case1 | | SSPE_case2 | |
| --- | --- | --- | --- | --- |
| **Data collection** |  | |  | |
| Microscope | Titan Krios  300  Falcon4  96,000  40  -1.0 to -2.0  0.824 | | Titan Krios  300  Falcon4  96,000  40  -1.0 to -2.0  0.824 | |
| Voltage (kV) |  |  |  |  |
| Detector |  |  |  |  |
| Magnification |  |  |  |  |
| Electron exposure (e–/Å^2^) |  |  |  |  |
| Defocus range (μm) |  |  |  |  |
| Pixel size (Å) |  |  |  |  |
| **Data processing** | CTE type I | CTE type II | CTE type I | CTE type II |
| Box size (pixel) | 400 | 400 | 300 | 300 |
| Symmetry imposed | C1 | C1 | C1 | C1 |
| Initial nr. filament segments | 342,441 | | 388,755 | |
| Final nr. filament segments | 222,257 | 17,756 | 348,830 | 32,882 |
| Map resolution (Å)  FSC threshold 0.143 | 2.3 | 5.1 | 3.0 | 3.7 |
| Helical rise (Å) | 2.38 | 2.39 | 2.37 | 2.37 |
| Helical twist (°) | 179.35 | 179.35 | 179.41 | 179.39 |
| **Refinement** |  |  |  |  |
| Model resolution (Å)  FSC threshold 0.5 | 2.9 |  |  | 3.8 |
| Map sharpening *B* factor (Å^2^) | -44 |  |  | -111 |
| Model composition  Non-hydrogen atoms  Protein residues  Ligands | 2870  375  0 |  |  | 3444  450  0 |
| *B* factors (Å^2^)  Protein | 136.7 |  |  | 259.5 |
| R.m.s. deviations  Bond lengths (Å)  Bond angles (°) | 0.007  1.43 |  |  | 0.009  1.746 |
| Validation  MolProbity score  Clashscore  Poor rotamers (%) | 1.38  1.37  1.52 |  |  | 2.49  8.40  4.55 |
| Ramachandran plot  Favored (%)  Allowed (%)  Disallowed (%) | 94.52  5.48  0 |  |  | 90.41  9.59  0 |
| EMDB | EMD-16532 |  |  | EMD-16535 |
| PDB | 8CAQ |  |  | 8CAX |

**Figure S1.**

**Cryo-EM 2D classifications and Fourier shell correlation (FSC) curves.**

(a), Representative 2D classification images of tau filaments from both cases of SSPE. Type I and Type II filaments of CTE are in evidence. Scale bar, 10 nm.

(b,c), Solvent-corrected FSC curves of cryo-EM half maps (left panel) and model to map validation (right panel). Type I filaments from SSPE case 1 are shown in (b), Type II filaments from case 2 are shown in (c). FSC curves between a model refined in half map 1 and half map 1 are shown in brown (model 1 vs half map 1); FSC curves between the same model and half map 2 are shown in green (model 1 vs half map 2).
